# The potential ameliorative impacts of cerium oxide nanoparticles against fipronil-induced hepatic steatosis

**DOI:** 10.1038/s41598-020-79479-5

**Published:** 2021-01-14

**Authors:** Lamiaa Wasef, Atef M. K. Nassar, Yasser S. El-Sayed, Dalia Samak, Ahmed Noreldin, Norhan Elshony, Hamida Saleh, Yaser H. A. Elewa, Shaimaa M. A. Hassan, Abdullah A. Saati, Helal F. Hetta, Gaber El-Saber Batiha, Masakazu Umezawa, Hazem M. Shaheen

**Affiliations:** 1 Department of Pharmacology and Therapeutics, Faculty of Veterinary Medicine, Damanhour University, Damanhour, 22511 ElBeheira Egypt; 2 Pesticides Chemistry and Toxicology, Plant Protection Department, Faculty of Agriculture, Damanhour University, Damanhour, Egypt; 3 Department of Veterinary Forensic Medicine and Toxicology, Faculty of Veterinary Medicine, Damanhour University, Damanhour, Egypt; 4 Department of Histology and Cytology, Faculty of Veterinary Medicine, The Scientific Campus, Damanhour University, Damanhour, Egypt; 5 Department of Histology and Cytology, Faculty of Veterinary Medicine, Zagazig University, Zagazig, Egypt; 6 Laboratory of Anatomy, Department of Biomedical Sciences, Graduate School of Veterinary Medicine, Hokkaido University, Sapporo, Hokkaido 060-0818 Japan; 7 Histology and Cell Biology Department, Faculty of Medicine, Menoufia University, Shebeen El-Kom, Egypt; 8 Department of Histology, College of Medicine, Batterjee Medical College, Aseer, Saudi Arabia; 9 Department of Community Medicine & Pilgrims Healthcare, Faculty of Medicine, Umm Al-Qura University, Mecca, 24382 Saudi Arabia; 10 Department of Medical Microbiology and Immunology, Faculty of Medicine, Assiut University, Assiut, 71515 Egypt; 11 Department of Materials Science and Technology, Faculty of Industrial Science and Technology Soga Laboratory, Tokyo University of Science, 9th Floor, Research Labs Building, 6-3-1 Niijuku, Katsushika, Tokyo 125-8585 Japan

**Keywords:** Chemical biology, Gastroenterology, Medical research

## Abstract

Fipronil (FIP) is a phenylpyrazole insecticide that is commonly used in agricultural and veterinary fields for controlling a wide range of insects, but it is a strong environmentally toxic substance. Exposure to FIP has been reported to increase the hepatic fat accumulation through altered lipid metabolism, which ultimately can contribute to nonalcoholic fatty liver disease (NAFLD) development. The present study aimed to examine the function of cerium oxide nanoparticles (CeNPs) in protecting against hepatotoxicity and lipogenesis induced by FIP. Twenty-eight male albino rats were classified into four groups: FIP (5 mg/kg/day per os), CTR, CeNPs (35 mg/kg/day p.o.), and FIP + CeNPs (5 (FIP) + 35 (CeNPs) mg/kg/day p.o.) for 28 consecutive days. Serum lipid profiles, hepatic antioxidant parameters and pathology, and mRNA expression of adipocytokines were assessed. The results revealed that FIP increased cholesterol, height-density lipoprotein, triacylglyceride, low-density lipoprotein (LDL-c), and very-low-density lipoprotein (VLDL-c) concentrations. It also increased nitric oxide (NO) and malondialdehyde (MDA) hepatic levels and reduced glutathione peroxidase (GPx) and superoxide dismutase (SOD) enzyme activities. Additionally, FIP up-regulated the fatty acid-binding protein (FABP), acetyl Co-A carboxylase (ACC1), and peroxisome proliferator-activated receptor-alpha (PPAR-α). Immunohistochemically, a strong proliferation of cell nuclear antigen (PCNA), ionized calcium-binding adapter molecule 1 (Iba-1), cyclooxygenase-2 (COX-2) reactions in the endothelial cells of the hepatic sinusoids, and increased expression of caspase3 were observed following FIP intoxication. FIP also caused histological changes in hepatic tissue. The CeNPs counteracted the hepatotoxic effect of FIP exposure. So, this study recorded an ameliorative effect of CeNPs against FIP-induced hepatotoxicity.

## Introduction

Around the world, pesticides are widely used to control plant and animal pests but they have direct and/or indirect toxic effects on beneficial organisms^1^. Among pesticides, fipronil (FIP) [5-amino-3-cyano-1-(2,6-dichloro-4-trifluoromethylphenyl) 4-fluoromethylsulfinyl pyrazole] is a second-generation phenylpyrazole insecticide^2^ extensively used in the veterinary clinical field against many pests, and ectoparasites^3,4^. It blocked the chloride channels regulated by GABA, resulting in an uncontrolled central nervous system (CNS) hyperexcitation and death^5^. Fipronil toxic effect depends on the disturbance of the antioxidant and oxidant system balance through the over-accumulation of reactive oxygen species (ROS) in the cells^6^, especially of liver tissue, which is the main organ for insecticides-detoxification^7^. This balance is a good indicator of exposure to toxic chemicals in hepatocytic mitochondria and endoplasmic reticulum^8^. Antioxidant/oxidant imbalance might lead to chemical changes in proteins and fat molecules structure resulting in the activation of lipid peroxidation (LPO) process^9^ and is often believed to enhance the hepatic cells oxidative injury^10^.

The accumulation of lipids in hepatic cells is more common in cases of exposure to toxic chemicals^11^ and leads to hepatitis and fibrosis^11^. Increased synthesis of triglycerides results in increased fatty acids (FA) accumulation in the liver. Consequently, mitochondrial oxidation of the free fatty acids (FFAs) damages the liver tissues, resulting in infiltration of neutrophil with subsequent ROS release and exacerbation of inflammatory reactions^12,13^. Alongside, cerium oxide nanoparticles (CeNPs) are among the most crucial metal-oxide nanoparticles, which play a technologically important role in synthesizing different industrial materials including polishing materials in the glass and optics industry, oxygen sensors, and ultraviolet filters^14^. CeNPs have gained much interest in biological applications due to their antioxidant properties. Based on the Ce^3+^/Ce^4+^ratio in CeNPs surface, this antioxidant activity will be as follows: superoxide dismutase (SOD) mimetic activity^15^, catalase (CAT) mimetic activity, hydroxyl and nitric oxide (NO) scavenging property^16^. Therefore, CeNPs have been used in medicine^15^ and pharmacy^17^ due to their potent antioxidant activity in scavenging free radicals produced in the process of oxidative damage. Reducing ROS production could reduce inflammation and consequent tissue injury^18^. No earlier studies explained the correlation between CeNPs and FIP, thus the objective of the existing study was to examine the ameliorative impacts of CeNPs on hepatic tissue against sub-acute toxicity caused by FIP administration in male albino rats.

## Materials and methods

### Chemicals

Cerium oxide nanopowder < 25 nm particle size, (CAS Number: 1306-38-3) was purchased from Sigma-Aldrich Co., USA. The CeNPs were suspended in demineralized water at a 35 mg/kg bwt concentration. Fipronil solution (5 mg/kg bwt) was prepared by dissolving the commercial product FIPROGENT 80% (MAC-GmbH, Sigmarszell, Germany) in demineralized water.

### Experimental animals

Twenty-eight healthy adult male albino rats (average weight 180 ± 10 g) were obtained from the Animal Breeding Unit, Faculty of Agriculture, Alexandria University. The animals were housed under a pathogen-free environment with controlled humidity, temperature (22 °C) and a 12 h light/dark cycle. The animal experiments were performed according to the Laboratory Animals of the National Institutes of Health (NIH) Care and Use Guidelines, and the study protocol was approved by the local authorities of Damanhour University, Egypt (DMU-2019-0023). Two weeks before the experiment was conducted, the animals were allowed to acclimatize the testing facility condition. Afterward, the rats were caged equally into four experimental groups, each consisting of seven rats. Group 1—control group (CTR) rats were orally received saline. Group 2—animals have orally received FIP solution (5 mg/kg bwt, 1/20 of the LD_50_)^19,20^. Group 3—the rats have orally received CeNPs solution (35 mg/kg body weight)^21^. Group 4—animals orally received FIP (5 mg/kg) and CeNPs (35 mg/kg) solutions using gastric tube daily. During the experimental period, rats were daily observed for any abnormal behavior and clinical signs.

### Blood and tissue sampling

On the 28th day of the experiment, all rats were prohibited from feeding overnight, weighed individually, and euthanized using an anesthesia system containing xylazine and ketamine HCl. Blood was gathered from the aortic vein, kept in anticoagulant-free test tubes, and the serum was isolated and kept at − 20 °C until lipid profile determination. The liver was excised and rinsed using physiological saline (NaCl 0.9%), wiped using filter paper and split into two sections – the first section kept rapidly at − 80 °C for biochemical and gene expression levels and the second section was used for histopathological and immunohistochemical examination after fixing in four percent paraformaldehyde (PFA) diluted in phosphate buffer saline (PBS) solution.

### Serum biochemical test

For examination of the serum lipid profile of the treated rats; triacylglycerol, cholesterol, HDL-c, LDL-c, and VLDL-c commercial kits were used, which were purchased from Bio-Diagnostics Co., Cairo, Egypt. The experiments were conducted following the manufacturer's instruction guidelines.

### Oxidant/antioxidant parameters in liver tissue homogenates

Hepatic tissues were moistened using phosphate-buffered saline (PBS, 0.1 M, pH 7.4). Using a disposable homogenizer (Biomasher; Nippi, Inc., Tokyo, Japan), homogenates of ten percent from the hepatic tissues were then prepared in cold potassium phosphate buffer (50 mM, pH 7.5). The resulted homogenates were centrifuged at 4 °C for 15 min at 10,000×*g*. The supernatants were used for evaluating lipid peroxidation indicators: NO and malondialdehyde (MDA)^22^ and antioxidant biomarkers: glutathione peroxidase (GPx) and SOD activity^23,24^ following the manufacturers' guidelines (Bio-diagnostics CO., Cairo, Egypt).

### Hepato-histological examination

The fixed samples were treated using the traditional paraffin embedding method, including drying in ascending grades of ethanol, disinfected in three changes of xylene and melted paraffin finished by inserting it at 65 °C in paraffin wax. Hematoxylin and eosin (H&E) were used to stain four µm thick sections as previously detected by Bancroft and Layton^25^ and Periodic acid Schiff (PAS) based on Layton and Bancroft^26^. Semi-quantitative scoring of cardiac lesions was calculated based on Gibson-Corley, et al.^27^. Briefly, the lesions in 10 fields were collected and summed randomly from each slide for each rat. The lesions were blindly recorded (Score scale: 0 = normal; 1 ≤ 25%; 2 = 26–50%; 3 = 51–75%; 4 = 76–100%).

### Immunohistochemical examination

Working dilutions, sources, methods, and antibodies for antigen recovery were listed in Table 1. The immunohistochemical technique in liver sections was investigated based on the method identified by Noreldin, et al.^28^ and Noreldin, et al.^29^. Briefly, the paraffin sections were prepared with a thickness of 4 µm, deparaffinized by xylene and re-moistened in graded alcohol and washed with distilled water. Afterward, endogenous peroxidase was deactivated by 3% H_2_O_2_ in absolute methanol for 30 min at 4 °C and washed again using PBS, blocking the nonspecific reaction at room temperature with 10% normal blocking serum for 60 min. Then, the sections were incubated overnight at 4 °C with the primary antibodies, washed with PBS, incubated for 60 min with biotin-conjugated goat anti-rabbit IgG antiserum or rabbit anti-goat IgG antiserum (Histofine kit, Nichirei Corporation) according to the species' primary antibody hosted. Then the sections were incubated for 30 min with streptavidin-peroxidase conjugate (Histofine kit, Nichirei Corporation) after washing with PBS. The streptavidin–biotin complex was further incubated for 3 min with a solution of 3,3′-diaminobenzidine tetrahydrochloride (DAB)-H_2_O_2_, pH 7.0. Finally, the sections were rinsed with distilled water and used Mayer's hematoxylin counterstain. A digital camera (Leica EC3, Leica, Germany) connected to a microscope (Leica DM500, Leica, Germany) was used to capture micrographs of the prepared sections. ImageJ software (National Institutes of Health, Bethesda, MD, USA) was used for immunostaining intensities’ quantification^30^. The mean of the inverse density of 10 randomly selected fields from different parts of 8 rats in each group was calculated byVis et al.^31^.

**Table 1:** List of antibodies, sources, working dilutions, and methods for antigen retrieval.

Antibody	Source	Dilution	Antigen retrieval	Heating condition
Rabbit polyclonal anti-Caspase3	(9662, Cell Signaling Technology, Danvers, Ma, USA)	1:300	10 mM citrate buffer (pH 6.0)	105 °C, 20 min
Rabbit polyclonal anti-Iba-1	(019-19741, Wako Osaka, Japan)	1:1200	10 mM citrate buffer (pH 6.0)	105 °C, 20 min
Goat polyclonal anti-PCNA	(sc-9857, Santa Cruz Biotechnology, CA, USA)	1:2000	Dako, 105 °C, 20 min	105 °C, 20 min
Rabbit polyclonal anti- Bax	(PU347-UP, San Ramon, Ca, USA)	1:30	None	None
Rabbit monoclonal anti-Cox-2	(RM-9121-S0, Thermo Fisher Scientific, Fremont, CA, USA)	1:100	10 mM citrate buffer (pH 6.0)	105 °C, 20 min

### Quantitative reverse transcription-polymerase chain reaction (RT-qPCR)

RNA-Spin^tm^ total RNA extraction kits (Cat. #17211) (INTRON biotechnology Inc. was used to extract total RNA from the liver tissues. Total RNA (1 mg) was used as a template for the first strand of complementary DNA (cDNA) using Maxima First Strand cDNA synthesis Kits from iNtRON Biotechnology Inc (Cat. #EZ00SS). Quantitative RT-PCR was conducted using Thermo Scientific Maxima SYBR Green/ROX qPCR PreMix kits from iNtRON Biotechnology Inc (Cat. #RT500S). The primers were acetyl-CoA carboxylase alpha *Rattus norvegicus*, peroxisome proliferator-activated receptor alpha (PPAR-α), Fabp1, *Rattus norvegicus* caspase3, and *Rattus norvegicus* Bcl-2 like protein (Table 2). The values of target genes have been standardized to household expression level, GAPDH.

**Table 2:** Primer sequences and accession number used for RT-qPCR.

Primer name	Accession number	Sequences
Rattus norvegicus acetyl-CoA carboxylase alpha (Acaca), mRNA	NM_022193.1	F: GTTGGACAACGCCTTCACAC R: GCGCATGGAATGGCAGTAAG
Rattus norvegicus fatty acid binding protein 1 (Fabp1), Mrna	NM_012556.2	F: AGGACCTCATCCAGAAAGGGA R: TGACCTTTTCCCCAGTCATGG
Rattus norvegicus peroxisome proliferator activated receptor alpha (Ppara), mRNA	NM_013196.1	F: ACCTTGTGCATGGCTGAGAA R: CCTTGGCAAATTCCGTGAGC
Rattus norvegicus Caspase3	NM_001284409.1	F: AGTTGGACCCACCTTGTGAG R: AGTCTGCAGCTCCTCCACAT
Rattus norvegicus Bcl-2 like protein 4	NM_007527.3	F: CACCAGCTCTGAACAGATCATGA R: TCAGCCCATCTTCTTCCAGATGGT

### Statistical analysis

Data were described as mean ± SEM. Results were analyzed statistically by one-way (ANOVA) test using the Statistical Analysis System (SAS) software version 13.2 (SAS, 2016). Considerable means have been compared with multiple reference checks of Tukey’s post-hoc. Results at *P* < 0.05 were considered statistically significant.

## Results

### Signs of toxicity

No obvious clinical signs or symptoms noticed all over the experimental period on rats that were exposed to FIP or/and CeNPs.

### Serum biochemical findings

The FPN-intoxicated group showed significant elevation (*P* < 0.05) in the serum levels of cholesterol (19.0%), TAG (25.2%), LDL-c (66.0%) and HDL-c (47.6%) when compared with the CTR one (Table 3). Meanwhile, rats treated with CeNPs and FIP + CeNPs exhibited remarkable reductions (*P* ≤ 0.05) in serum cholesterol levels by 8.5 and 9.3%, respectively. Rats treated with CeNPs and FIP + CeNPs had similar content of TAG, VLDL-c, LDL-c, and HDL-c to that of the CTR group. The VLDL-c levels were not different in the serum of all treated groups (Table 3).

**Table 3:** Effects of Fipronil and CeNPs intake on Serum lipid profile indices in rats.

Group	TAG (mg/dL)	VLDLc (mg/dL)	LDLc (mg/dL)	Cholesterol (mg/dL)	HDLc (mg/dL)
CRT	70.4125 ± 0.38534^b^	14.1825 ± 0.78979^a^	39.9725 ± 2.64559^b^	115.0000 ± 0.40825^b^	45.3500 ± 1.28225^b^
FIP	88.2000 ± 1.37174^a^	15.9900 ± 1.01985^a^	66.0900 ± 2.70945^a^	137.1250 ± 1.63777^a^	66.8750 ± 1.73656^a^
CeNPs	66.0250 ± 0.83902^b^	13.2175 ± 0.16815^a^	31.3875 ± 2.64483^b^	105.2125 ± 0.79441^c^	41.7750 ± 1.18348^b^
FIP + CeNPs	65.8125 ± 1.31694^b^	14.4725 ± 1.06092^a^	34.3950 ± 2.84348^b^	104.3425 ± 1.55696^c^	46.8500 ± 1.73133^b^

The effect of cerium oxide nanoparticles (CeNPs) on serum lipid profile concentrations in FIP intoxicated rats. All values are expressed as the mean ± SE, n = 7. Means with different superscript letters (a, b, c) are statistically significant at (p ≤ 0.05). *FIP* Fipronil, *CeNPs* cerium oxide nanoparticles, *TAG* triacylglycerides, *VLDLc* very-low density lipoproteins, *LDLc* low density lipoproteins, *HDLc* high density lipoproteins. Values with different superscript letters within the same column are significantly different (*P* ≤ 0.05, One-way ANOVA with Tukey’s HSD post hoc test).

### Liver lipid peroxidation and antioxidative indices

The results presented in Table 4 revealed that the FIP-treated group showed substantial increase (*P* ≤ 0.05) in MDA (68%) concentrations and decrease in GPx (39%) and SOD (46%) enzymes activities in the liver tissue in relative to the CTR one. The concentrations of MDA in liver tissue (37%) in CeNPs-treated rats was reduced significantly (*P* ≤ 0.05), while GPx and SOD enzyme activities were similar to normal CTR values (Fig. 2). Concentrations of serum NO were nearly comparable in CTR, CeNPs, and FIP + CeNPs groups. The FIP toxic effects on hepatic GPx, MDA, SOD, and NO were substantially reduced (*P* ≤ 0.05) by CeNPs administration indicating the effect of CeNPs in alleviating oxidative damage caused by FIP.

**Table 4:** Effects of Fipronil and CONPs intake on hepatic oxidative/antioxidative indices in rats.

Group	MDA (nmol/mg)	NO (µmol/mg)	GPx (U/mg)	SOD(U/mg)
CRT	0.3825 ± 0.00479^b^	56.5825 ± 1.14776^bc^	42.4325 ± 2.19678^a^	7.7000 ± 0.48642^a^
FIP	0.6475 ± 0.02016^a^	86.5925 ± 0.50688^ab^	25.6025 ± 1.30649^b^	4.1650 ± 0.26247^b^
CeNPs	0.2375 ± 0.00750^c^	49.8525 ± 1.33769^c^	45.9525 ± 1.16427^a^	9.1800 ± 0.47450^a^
FIP + CeNPs	0.4250 ± 0.01708^b^	53.8875 ± 1.99681^bc^	41.9800 ± 1.12886^a^	8.1450 ± 0.27834^a^

The effect of cerium oxide nanoparticles (CeNPs) on liver tissue lipid peroxidation and activities of antioxidant enzymes in FIP intoxicated rats All values are expressed as the mean ± SE, n = 7. Means with different superscript letters (a, b, c) were significantly different at (P ≤ 0.05). *FIP* Fipronil, *CeNPs* cerium oxide nanoparticles, *MDA* Malondialdehyde, *SOD* Superoxide dismutase, *GPx* Glutathione peroxidase, *NO* Nitrogen oxide. Values with different superscript letters within the same row are significantly different (*P* ≤ 0.05, One-way ANOVA with Tukey’s HSD post hoc test).

### Histopathological investigation of hepatic tissue

**Figure 1:**
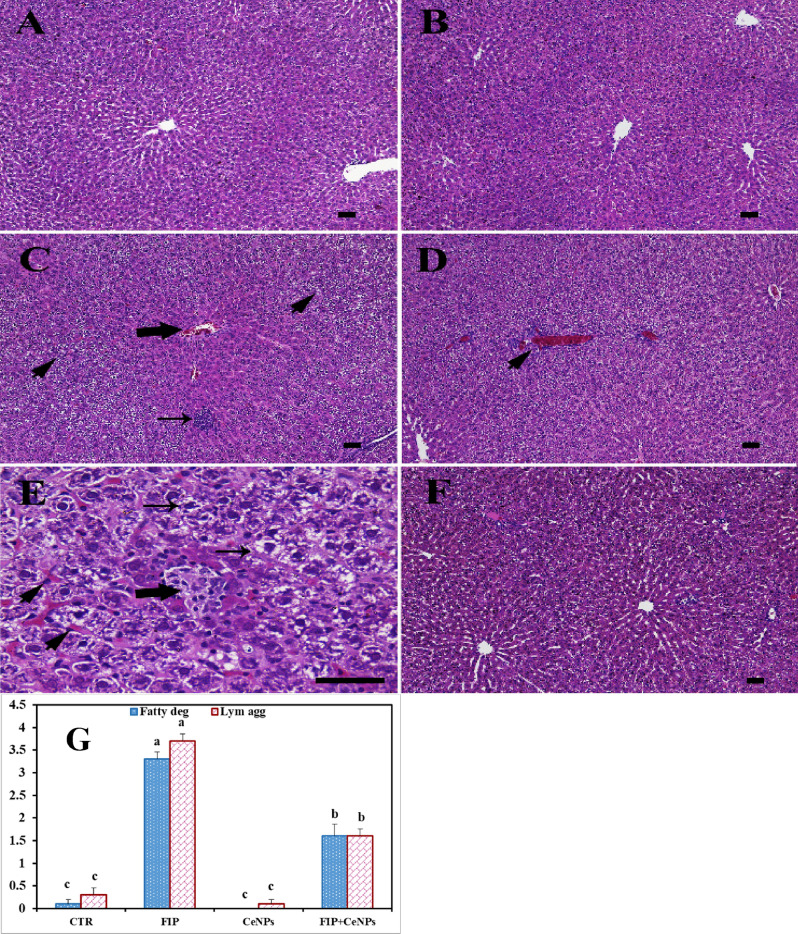
Histopathological examination of rat liver. (**A**) Negative control group. (**B**) CeNPs group. (**C**–**E**) FIP group showing in (**C**) congested central vein (arrow), massive fatty degeneration in the periportal areas (arrowheads) and lymphocytic aggregation (arrow) in between massive fatty degeneration (arrowheads). (**D**) Highly congested portal vein surrounded with lymphocytic infiltration (arrowhead). (**E**) Necrotic foci (thick arrow), congested liver sinusoids (arrowheads) and fatty degeneration (thin arrows). (**F**) FIP group that treated with CeNPs. Scale bar = 50 µm. (**G**) H&E semi quantitative scoring of hepatic fatty degeneration and lymphocytic aggregations. Data expressed as Mean ± SE, analyzed using one-way ANOVA at *P* ≤ 0.05, column with different letters (a, b & c) indicate significant difference among the values of different groups.

The histological findings revealed a polygonal hepatocyte producing substantial anastomosis plaques in the liver tissue in the CTR animals with acidophilic cytoplasm (Fig. 1A). Hepatic cells exhibited one or two large central round heavy hematoxylin-stained nuclei. Moreover, one or more nucleoli were detected in some hepatic cells (Fig. 1A). Hepatocyte plates and the hepatic capillaries barriers (sinusoids) laminated the Disse’s space, while hepatic capillary walls were bordered by Kupffer cells (Fig. 1A) and distinguished primarily by elongated and heavily stained nuclei. The tissues of the CeNPs group didn’t show any histopathological effects (Fig. 1B). In contrast, the FIP group revealed portal vein congestion surrounded with lymphocytic infiltration, lymphocytic aggregation in between massive fatty degeneration, congested central vein, necrotic foci, and nuclear condensation (Fig. 1C–E). It was noticed that CeNPs protected hepatic cells against FIP adverse effects, where it restored the normal architecture of liver tissues (Fig. 1F). The FIP treatment significantly increased the fatty bodies’ degeneration and lymph aggregation compared to CeNPs and the CTR group, while CeNPs significantly countered these effects (Fig. 1G).

**Figure 2:**
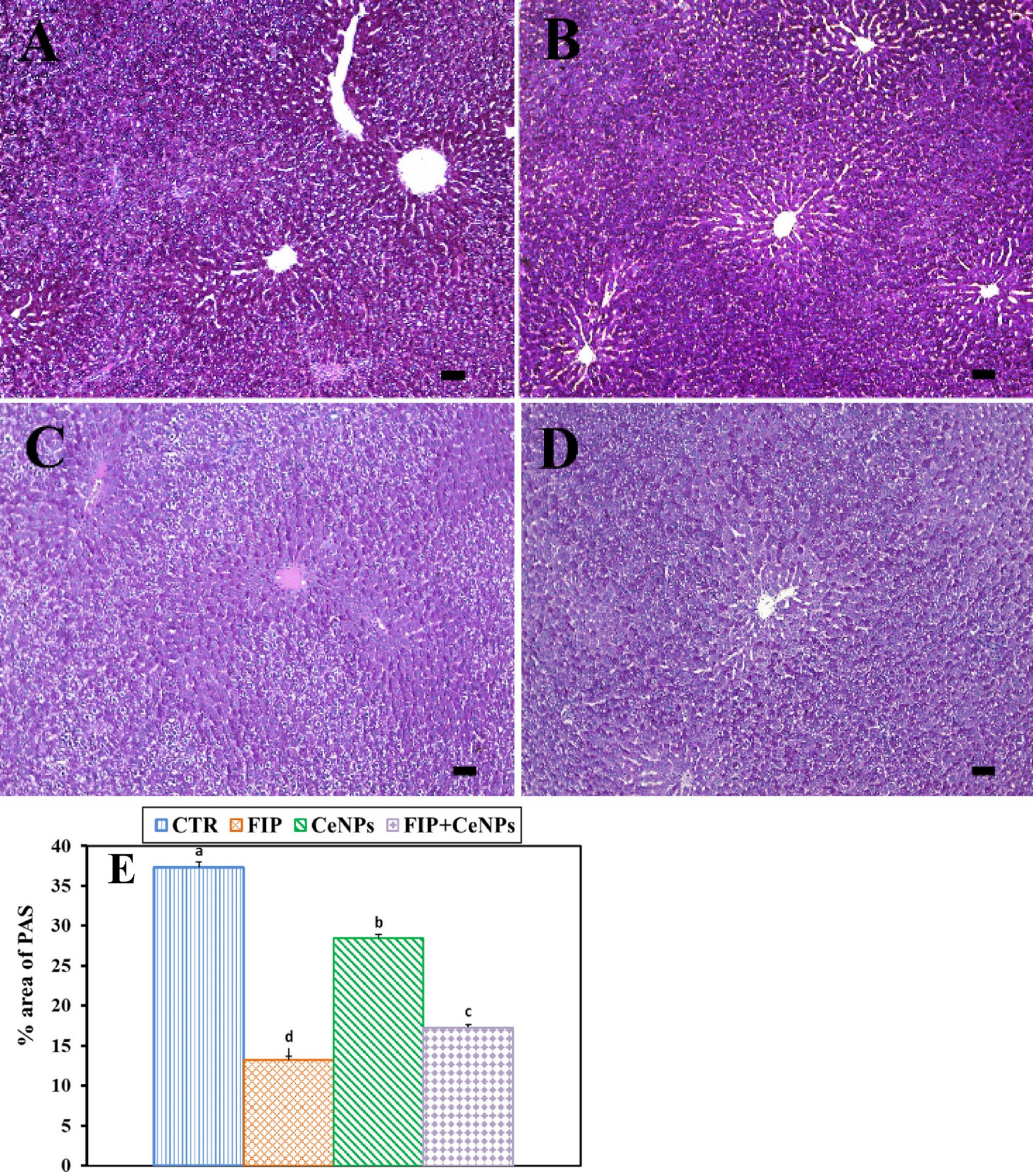
Histochemical staining of rat liver by periodic acid Schiff (PAS). (**A**) Negative control group. (**B**) CeNPs group. (**C**) FIP group. (**D**) FIP group that treated with the CeNPs group. Scale bar = 50 µm. (**E**) Quantification of PAS in the hepatic tissues in different groups Data expressed as Mean ± SE, analyzed using one-way ANOVA at *P* ≤ 0.05, column with different letters (a, b, c, & d) indicate significant difference among the values of different groups.

CeNPs groups had the greatest PAS distribution in all hepatocytes (Fig. 2A,B). Also, FIP caused a weak and uneven distribution of PAS and decreased glycogen content (Fig. 2C) in relation to the CTR one. Additionally, the FIP group that was treated with CeNPs had a moderate PAS reaction (Fig. 2D). FIP showed a significant decrease in PAS distribution concerning CTR and the CeNPs-treated group. CeNPs showed a high even distribution of PAS reaction in all hepatic lobules when combined with the FIP (Fig. 2E).

### Immunohistochemistry

**Figure 3:**
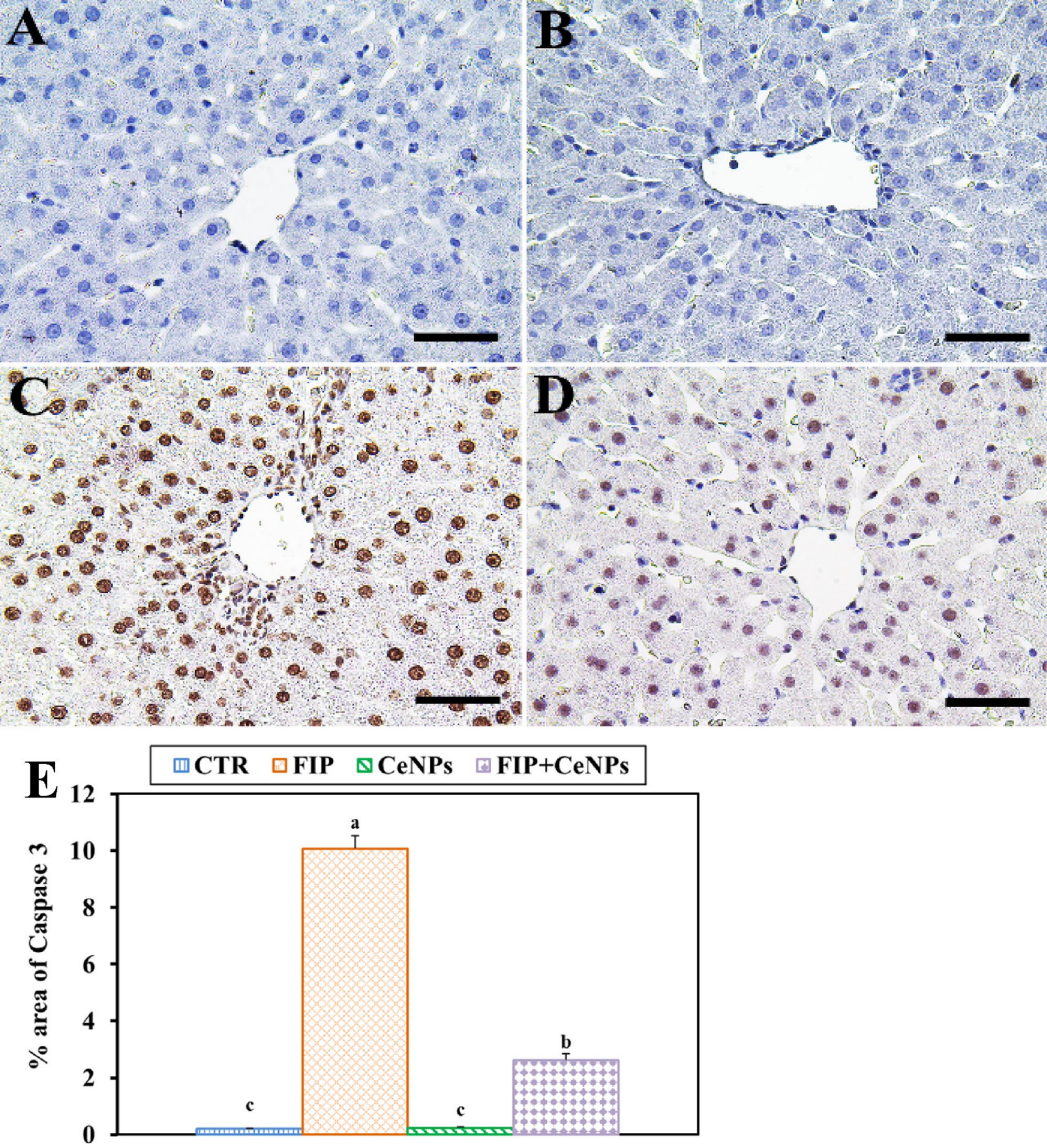
Immunohistochemical staining of rat liver by Caspase3. (**A**) Negative control group. (**B**) CeNPs group. (**C**) FIP group. (**D**) FIP group that treated with CeNPs. Scale bar = 50 µm. (**E**) Quantification of Caspase3 in the hepatic tissues in different groups. Data expressed as Mean ± SE, analyzed using one-way ANOVA at *P* ≤ 0.05, column with different letters (a, b & c) indicate significant difference among the values of different groups.

The FIP treatment showed a significant increase in caspase3 reaction concerning CTR- and CeNPs-treated groups. On the other hand, the FIP-treated group was protected with cerium as shown by a very weak caspase3 reaction (Fig. 3).

**Figure 4:**
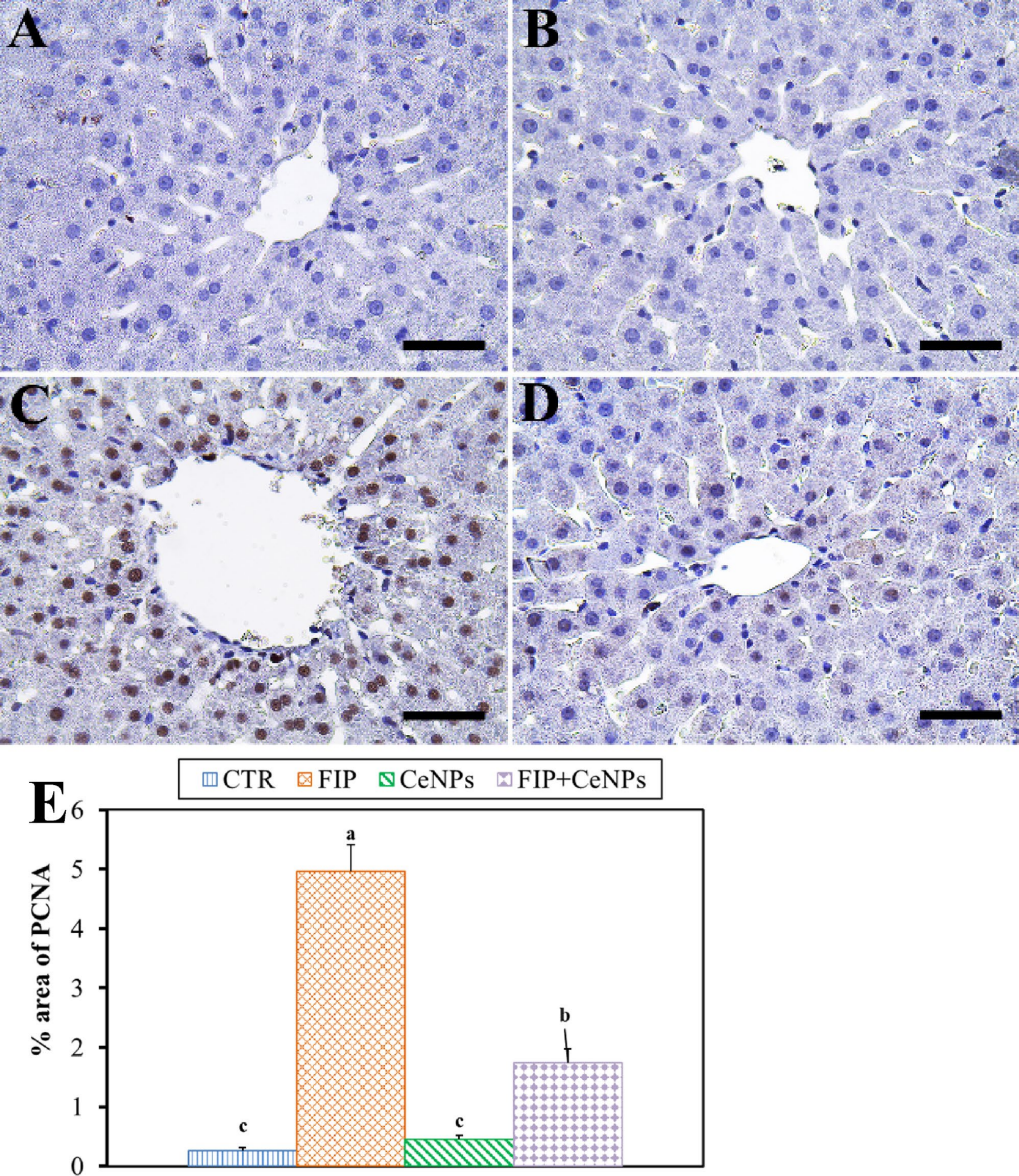
Immunohistochemical staining of rat liver by proliferating cell nuclear antigen (PCNA). (**A**) Negative control group. (**B**) CeNPs group. (**C**) FIP group. (**D**) FIP group that treated with CeNPs. Scale bar = 50 µm. (**E**) Quantification of PCNA in the hepatic tissues in different groups. Data expressed as Mean ± SE, analyzed using one-way ANOVA at *P* ≤ 0.05, column with different letters (a, b & c) indicate significant difference among the values of different groups.

Also, FIP-treated group showed strong PCNA reaction in the nuclei of most hepatocytes compared to CTR and CeNPs groups that showed negative PCNA reaction in the nuclei of hepatocytes, while FIP + CeNPs-treated group showed nearly negative PCNA reaction in nuclei of the hepatocytes (Fig. 4).

**Figure 5:**
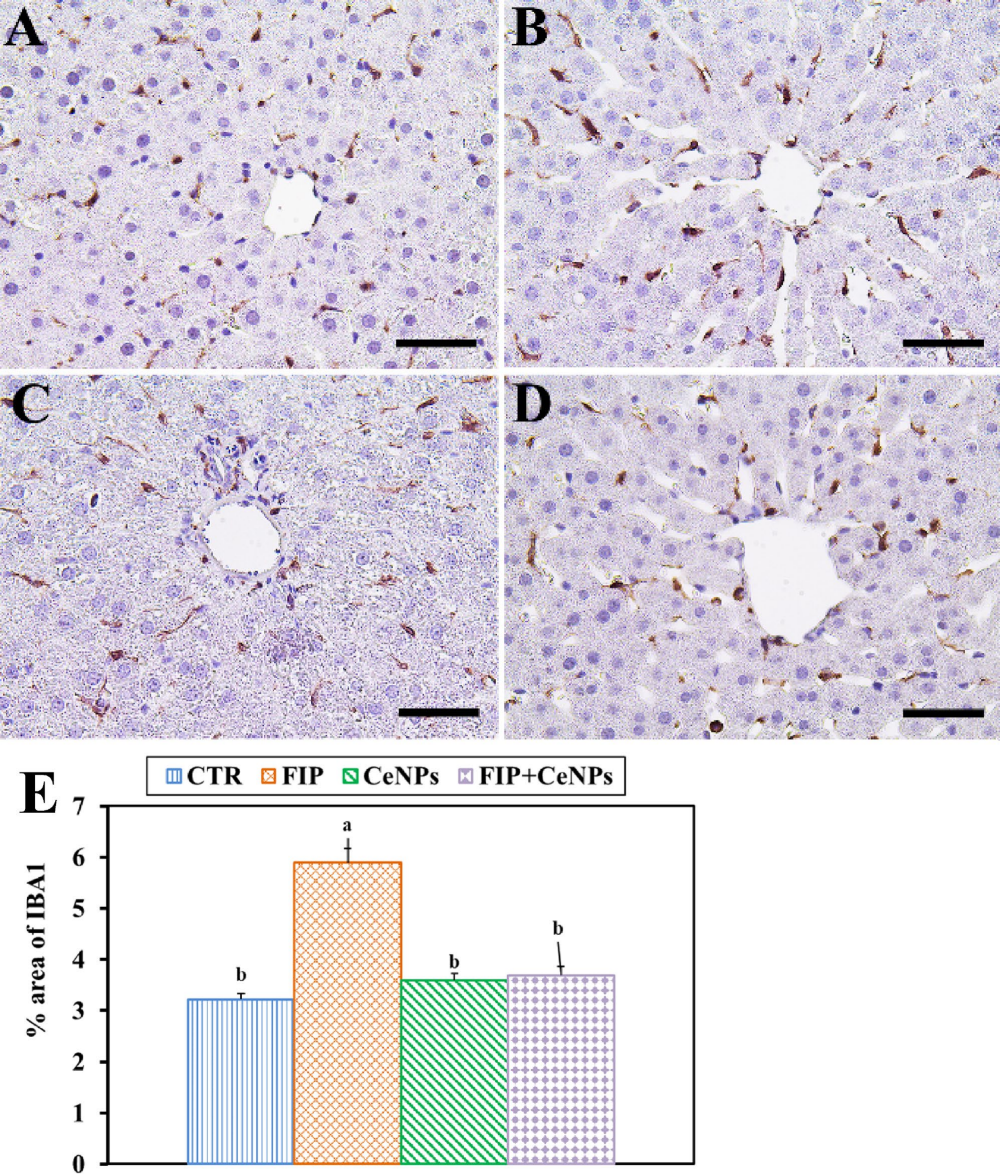
Immunohistochemical staining of rat liver by ionized calcium-binding adapter molecule 1 (Iba-1). (**A**) Negative control group. (**B**) CeNPs group. (**C**) FIP group. (**D**) FIP group that treated with CeNPs. Scale bar = 50 µm. (**E**) Quantification of I Iba-1 in the hepatic tissues in different groups. Data expressed as Mean ± SE, analyzed using one-way ANOVA at P ≤ 0.05, column with different letters (a & b) indicate significant difference among the values of different groups.

**Figure 6:**
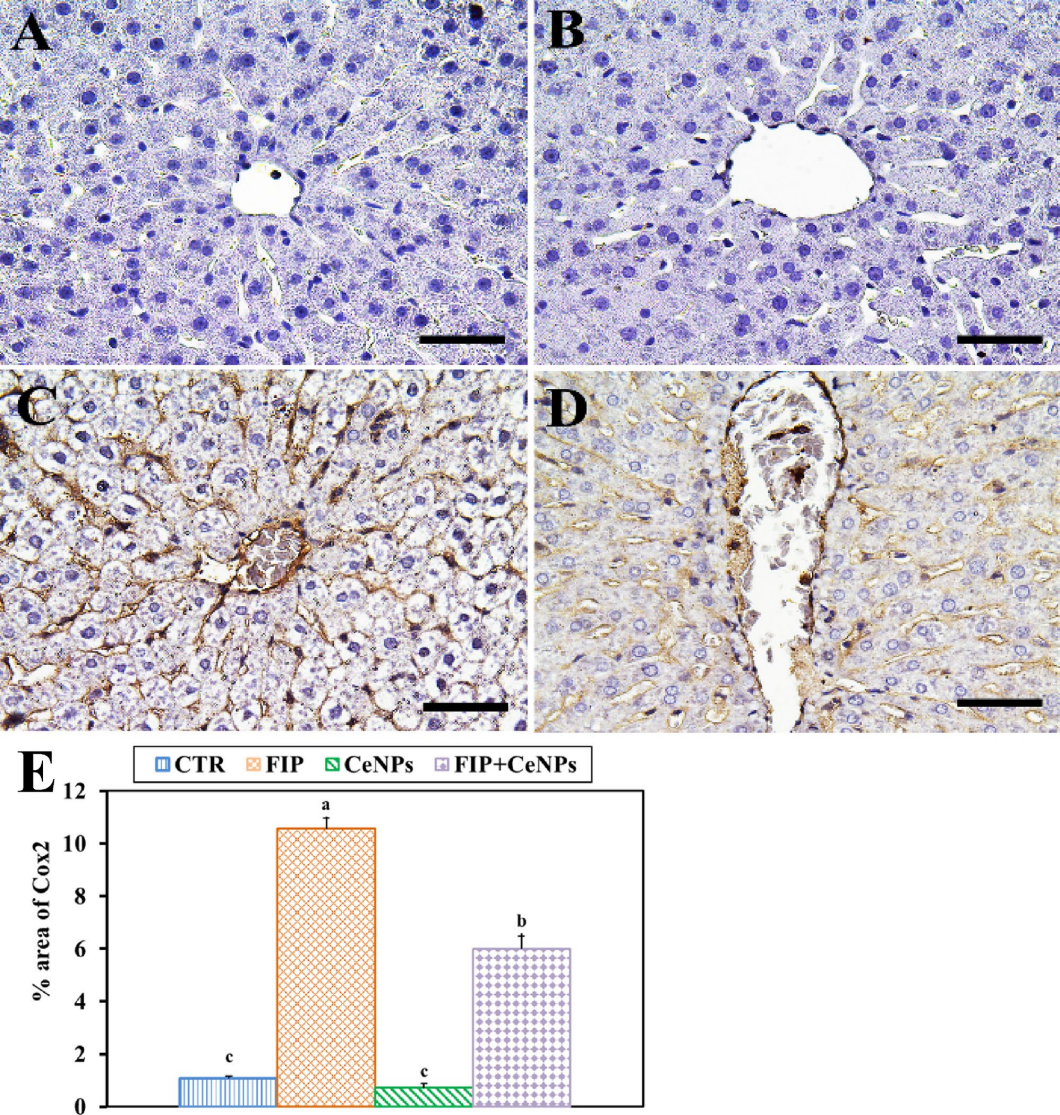
Immunohistochemical staining of rat liver by COX-2. (**A**) negative control group. (**B**) CeNPs group. (**C**) FIP group. (**D**) FIP group that treated with CeNPs. Scale bar = 50 µm. (**E**) Quantification of COX-2 in the hepatic tissues in different groups. Data expressed as Mean ± SE, analyzed using one-way ANOVA at *P* ≤ 0.05, column with different letters (a, b & c) indicate significant difference among the values of different groups.

Immunostaining of rat liver tissues by massive ionized calcium-binding adapter molecule1 (Iba-1) in the case of the FIP-intoxicated group showed a strong inflammatory process via strong cyclooxygenase (COX-2) reaction and Iba-1 positive hepatic macrophage in the hepatic sinusoids in comparison to fipronil group that treated with cerium nanoparticles. The latter group showed low IBA1 positive hepatic macrophage in the hepatic sinusoids. CTR and CeNPs groups showed normal distribution of the Iba-1positive hepatic macrophage in the hepatic sinusoids (Fig. 5) with a negative COX-2 reaction (Fig. 6). CeNPs attenuated FIP impacts on the endothelial cells of the hepatic sinusoids and showed a moderate reaction (Fig. 6).

### mRNA expression of FABP, ACC, PPAR-α, Caspase3, and Bcl-2

The results of mRNA expressions of FABP, ACC, PPAR-α, caspase3 and Bcl-2 genes in liver tissues were presented in Table 5. The results revealed up-regulation (*P* ≤ 0.05) in the comparative mRNA expressions of FABP, ACC, PPAR-α, and caspase3, while exhibited down-regulation in the Bcl-2 gene in the liver tissue of rats that received FIP when compared with the CTR animals. Meanwhile, CeNPs treatment down-regulated the mRNA expression of ACC and caspase3 and up-regulated the FABP, PPAR-α, and Bcl-2 compared to CTR. In the case of the FIP group that was treated with CeNPs, significant up-regulation in FABP, PPAR-α and Bcl-2 were observed. On the other hand, ACC and caspase3 mRNA expression were significantly down-regulated (Table 5).

**Table 5:** mRNA expression of fatty acid-binding protein.

Gene group	FABP	PPARα	ACC	Bcl2 like protein 4	Caspase3
FIP	2.230447721 ± 0.423	4.363039 ± 0.953	2.17347 ± 0.65	0.736624 ± 0.125	1.596966 ± 0.313
CeNPs	1.28877463 ± 0.329	1.395388 ± 0.351	0.076875 ± 0.021	1.670562 ± 0.346	0.240149 ± 0.051
FIP + CeNPs	1.428333957 ± 0.376	1.813781 ± 0.40459	0.846941 ± 0.203	1.081225 ± 0.165	0.82302 ± 0.143

The effect of cerium oxide nanoparticles (CeNPs) on liver mRNA expressions of FABP (fatty acid-binding protein), PPARα (peroxisome proliferator activated receptor- alpha), ACC (Acetyl COA Carboxylase), Bcl2 Like protein 4 and Caspase3 in FIP intoxicated rats. Data are expressed as Mean ± SE (n = 7). *FIP* Fipronil, *CeNPs* cerium oxide nanoparticles.

## Discussion

Hepatoxicity changes induced by FIP might be due to the imbalance of the antioxidant system, which might lead to the formation of ROS that activates the apoptotic process and disturbances in the lipid profile of rats^32,33^. Current results showed that CeNPs was a chemoprotective agent against FIP-induced hepatotoxicity. The hepatic cells were documented to have a significant and critical function in lipogeneses and glucose metabolism processes^34^. The disturbance of lipid and glucose metabolism might be observed by the excess accumulation of FA in the hepatic cells^35^. Consequently, increasing hepatic FA uptake^36^ leads to an increase in the hepatic triglyceride (TG) levels, which induces NAFLD development^37^. The changes in lipid profile might be caused by the accumulation of a high level of FA in circulation and hepatic lipogenesis, resulting in TG accumulation in the liver while dietary lipids represent a large percentage of intrahepatic lipids^38,39^.

The current study explained a decreased weight gain in CeNPs-treated rats relative to CTR ones. Also, the animals treated with FIP showed a remarkable elevation in serum lipid profile (TAG, HDL-c, LDL-c, and cholesterol) compared to CTR, CeNPs, and FIP + CeNPs groups. The significant modification in lipids might be due to the oxidative stress produced by FIP, which ultimately leads to liver inflammation and injury. These results indicated that FIP induced lipogenesis and lipid accumulation via increased expression of FABP and ACC1 in comparison to the CeNPs-treated group, which showed downregulation of ACC1 along with the up-regulation of FABP like the CTR groups. Activated AMPK (adenosine monophosphate-activated protein kinase) that phosphorylates the main proteins associated with controlling lipid and carbohydrate metabolism, followed by ATP inhibition that consumes anabolic pathways, such as cholesterol, isoprenoid, and FA synthesis, hepatic gluconeogenesis^40^ and induces lipolysis^41,42^. Some studies revealed that FIP increases adipogenesis by downregulating AMPKα, which disrupts lipid metabolism, which may demonstrate the high serum cholesterol level^20,33^. Fipronil insecticide has been reported to raise the levels of intracellular Ca^+2^ by altering the plasma membrane permeabilization^43^, leading to a disruption of adipogenesis and lipid metabolism process by the pathway mediated by Ca MKKβ and AMPK^44^.

FIP substantially elevated fatty acid synthase (FAS) and acetyl Co-A carboxylase (ACC) expression (the two rate-limiting enzymes for lipogenesis)^45^ when compared with CTR group. Likewise, FABP (a protein that transports FA in the cytoplasm for metabolic process or storage) expression^46^, was significantly enhanced by FIP therapy relative to CTR^45^. Regarding CeNPs, interestingly, Rocca et al.^47^ identified CeNPs as a new anti-obesity pharmaceutical formula after being tested in vitro and in vivo. They stated that CeNPs interfere with the adipogenic mechanism by decreasing the mRNA transcription of genes included in adipogenesis through inhibiting the accumulation of triglycerides found in 3T3-L1 pre-adipocytes. Intraperitoneal injection of CeNPs at a dose of 0.5 mg/kg did not show toxic symptoms in rats, however, a significant decrease in body weight and insulin, leptin, glucose, and TG plasma levels are observed in comparison to CTR group^47^. Our results are consistent with other studies that demonstrate the ability of CeNPs to activate AMPK which indicates inhibition of adipogenesis with decreased PPARαC/EBPα expression as well as adipogenic markers including ACC and FAS^48,49^.

Lipogenesis inhibition due to ACC phosphorylation may be the major regulatory step in FA synthesis and oxidation^50^. ACC stimulates the malonyl-CoA synthesis, which is a FA synthesis substrate and is inhibited by AMPK-mediated ACC phosphorylation^51^. Metabolism enhancement is considered as one of the anti-inflammatory actions of CeNPs mechanisms since that results reported herein have demonstrated that CeNPs substantially decreased the weight gain of rats, which might reduce the visceral obesity of rats (A. Rocca, S. Moscato, F. Ronca, S. Nitti, V. Mattoli, M. Giorgi, G. Ciofani, Pilot in vivo investigation of cerium dioxide nanoparticles as a novel anti-obesity pharmaceutical formulation, Nanomed. Nanotechnol., Biol. Med. 11 (7) (2015) 1725–1734). The mass of visceral adipose tissue in CeNPs-treated rats was approximately twice less than CTR^52^. PPARα is the most prevalent isotype in hepatic cells and participates in several lipid metabolism aspects^53^, such as FA synthesis, degradation, storage, transport, lipoprotein metabolism and ketogenesis throughout fasting. PPARα coordinates various de novo lipid synthesis pathways in the fed state to provide FA for hepatic TG storage during starvation times^54^. PPARα's mRNA expression in rats was the highest in tissues with high FA oxidation levels, such as kidney, heart, liver, and brown adipose tissue^55,56^. High-fat diet administration is often related to PPARα target genes hepatic expression participates in FA oxidation in wild-type mice, and it has been proposed the adaptive or protective effect of PPARα^57,58^. Few reports have investigated the outcomes of PPARα for treatments for NAFLD^59^. The improvement of β-FAO by PPARα agonist^60^ might protect the mice from induced liver injury^61^. Also, the recent study explained the high PPARα expression in FIP-treated rats that might be due to the increased level of FFAs and b-oxidation that usually caused by FFAs load in the mitochondria. This increases the load on the endoplasmic reticulum, which leads to ROS production causing oxidative stress and inflammatory pathway activation^62^. Besides its role in metabolism regulation, PPARα is also believed to have anti-inflammatory activity^63^.

There has been cumulative evidence that pesticide toxicity inhibits redox homeostasis as well as oxidative damage induction. Several studies suggested that redox homeostasis disturbance was triggered during FIP toxicity was because of high ROS production^33,64,65^. Hepatotoxicity induced by FIP in this study might be due to increased levels of MDA and NO (the indicator of LPO) because of the high reactive oxygen metabolites production especially the hydroxyl radicals^33,66^, LPO play role in the disruption of the integrity of cellular membranes and implicated in liver injuries^67^. ROS can target cell membranes and other cellular molecules, resulting in protein oxidation, lipid peroxidation, caspase3 activation, and DNA damage^33,68^, leading to cell dysfunction^69,70^. The protective mechanisms toward oxidative stress mainly through balances mediated by non-enzymatic and enzymatic antioxidants^71^. In current study, the decrease in SOD and GPx activities of rats subjected to FIP might be due to the excess production of O_2_ that is rapidly transformed by SOD and GPx to H_2_O_2_ and water, respectively as reported in the liver of pregnant rats and their offspring due to the inadequate ROS detoxification produced by FIP in hepatocyte^33,66^.

It was recognized that liver cells showed the most active absorption and retention of CeNPs stored in the liver for eight weeks at least^62,72,73^. Recently^62^, demonstrated that the antioxidant activity of CeNPs was through increasing the antioxidant enzymes (SOD and GPx) with decreasing the lipid peroxidation markers (MDA and NO). CeNPs have a high ability to eliminate free radicals as soon as they are produced during ROS imbalance as they can inversely transform between Ce^3+^ and Ce^4+^ found on the surface^74^. The lipid peroxidation reduction after CeNPs administration diminishes ROS harmful effects on the hepatic tissue. The mechanism of scavenging of ROS/RNS of CeNPs depends on the physicochemical properties, the ability of CeNPs to absorb and release oxygen^75^. CeNPs can act as a catalyst that mimics the antioxidant enzyme SOD^76^. So, there are two ways that CeNPs might act as an antioxidant against FIP toxicity. The first is linked to its antioxidant activity because the intensification of lipid peroxidation in the liver is among the causes of the inflammation, while the second is controlled by AMPK-PPAR-α–signaling mechanism^77^. The inflammatory processes involved in the pathogenesis of liver damage and obesity-related NAFLD^78^. Furthermore, fat destroys liver tissues and induces neutrophil infiltration by releasing ROS and aggravating inflammatory processes^12,13^. High accumulation of neutral lipid (mostly TG in hepatic cell lipid droplets) begins the early NAFLD pathological stages^79^. NAFLD’s pathogenesis is not well known but is suggested as a “two-hit” mechanism^80^. The first “hit” results in lipid aggregation and its mechanisms will likely include dysregulated lipid homeostasis such as de novo lipogenesis, β-oxidation, lipid storage and trafficking, and VLDL-c secretion^81^. This hepatic steatosis characterizes the liver to a “second hit” that results in inflammation, a primary pathophysiological symptom of steatohepatitis and advanced hepatic disorders^80,82^. Oxidative stress has been suggested as the main mediator of this “second hit”^80,83^. So, increased FFAs levels accompanied by PPAR-α stimulation, which in turn leads to ROSproduction and cell injury ^81^.

The efficiency of CeNPs on liver tissues was demonstrated by restoring the tissues architecture; necrosis, inflammation, and a decline of dystrophy in rats exposed to the FIP corroborated the histopathological lesions observed in the study^84^. FIP group revealing congested central vein, massive fatty degeneration in the periportal areas, lymphocytic aggregation in between massive fatty degeneration, with lymphocytic infiltration. Also, necrotic foci, congested liver sinusoids, and fatty degeneration might be due to that FIP increase lipid peroxidation. The FIP group that was protected with cerium nanoparticles showed normal liver architecture similar to the results of^72^. Studies showed an important connection between PCNA, Iba-1, and COX-2 expression and the inflammatory reaction as well as mitotic division^85^. Iba-1 participates in macrophage inflammatory pathways, including proliferation, migration, and signal transduction^86^. Proliferating cell nuclear antigen (PCNA) is believed to have an important role in controlling DNA synthesis as well as cell proliferation^87^. Also, COX isozymes (COX-1 and 2) are particularly important, as they are the main NSAIDs targets^88^, that parameters detected using the immunohistochemical.

In current study, Iba-1 cells were observed in the FIP-treated group with strong PCNA and COX-2 reaction concentrated in the nuclei of the most hepatocytes that may be due to FIP induced liver injury while in case of FIP group that protected by CeNPs show low Iba-1 positive hepatic macrophage and nearly negative PCNA and COX-2 reaction in nuclei of the hepatocytes, So, liver regeneration is the expected physiological response. Also, FIP induces apoptosis by strong caspase3 reaction in all nuclei of hepatocytes in opposite to FIP group previously protected with cerium showing very weak caspase3 reaction in the nuclei of hepatocytes. That might be due to the cytotoxic activity of fatty acid that influences cell survival. Long term accumulation of lipids may lead to hepatocyte necrosis or apoptosis^89^. MDA and NO interacted directly with the DNA and triggered DNA adducts and nuclear condensation that promoted apoptosis via cytochrome C and further caspase3 activations as detected by immunostaining, along with the induced mitochondrial dysfunction. Several reports revealed that FIP triggered cell death through apoptotic pathways^20^.

## Conclusions

In this study, FIP induced hepatotoxicity through disturbance in the serum lipid profile (VLDL-c, HDL-c, TAG, LDL-c) and cholesterol level might be due to increasing the mRNA fatty acid-binding protein expression and ACC genes and FIP can cause serious tissue injury in the liver caused by oxidative stress through increasing MDA and NO with decreasing SOD and GPx and apoptosis formation by increment caspase3 and decreasing BCL-2. While CeNPs could be used to activate the protective mechanisms against oxidative damage caused by FIP in the liver. Moreover, CeNPs effectively relieve the inflammatory processes in the blood of rats that may reduce obesity defects in liver damage.
